# Integrin β2 Plays a Significant Role in Therapeutic Angiogenesis Through Hematopoietic Stem Cell Transplantation

**DOI:** 10.3390/life15020195

**Published:** 2025-01-28

**Authors:** Orie Saino, Yuko Ogawa, Kazuta Yasui, Akihiro Fuchizaki, Rie Akamatsu, Yoriko Irie, Mitsunobu Tanaka, Takafumi Kimura, Akihiko Taguchi

**Affiliations:** 1 Department of Regenerative Medicine Research, Foundation for Biomedical Research and Innovation at Kobe, 2-2, Minatojima-Minamimachi, Chou-Ku, Kobe 650-0047, Hyogo, Japan; saino.orie@fbri.org (O.S.);; 2 Japanese Red Cross Kinki Block Blood Center, 7-5-17, Asagi Saito, Ibaraki 567-0085, Osaka, Japan; 3 Department of Preventive Medicine and Public Health, National Defense Medical College, 3-2, Namiki, Tokorozawa 359-8513, Saitama, Japan

**Keywords:** hematopoietic stem cell, cell therapy, adhesion factors, integrin β2, CD18, stroke

## Abstract

The efficacy of hematopoietic stem cell (HSC) therapy for cerebral infarction has been previously demonstrated. However, the lack of response in some patients has hindered its widespread use. To establish HSC therapy as a standard treatment, it is important to examine the causes of non-responsiveness. In this study, we aimed to identify the specifications of transplanted cells based on their therapeutic mechanisms to predict treatment success. We found that HSC therapy activates injured cerebral endothelial cells via gap junctions because cell adhesion between HSCs and the endothelium plays an essential role in cellular communication via gap junctions. The expression of the adhesion molecule integrin β2 (CD18) in CD34-positive (CD34^+^) cells was identified as critical for the therapeutic effect on cerebral infarction in a murine model. Cells with low CD18 expression exhibited a weaker therapeutic effect than cells with high CD18 expression, even when the same number of HSCs was administered. The expression of CD18 in CD34^+^ cells can be used as a specification marker for transplanted HSCs and is useful for identifying non-responders. Furthermore, quantification of CD18 expression is crucial for evaluating the cellular potential of cell-based therapies for diseases where therapeutic effects are mediated through cell adhesion.

## 1. Introduction

Stem cell therapy is a promising treatment for ischemic diseases, including stroke. Hematopoietic stem cells (HSCs) have shown a remarkable ability to promote angiogenesis in both nonclinical and clinical studies [1,2,3,4,5,6]. However, some patients do not respond to cell therapy, as both positive and neutral/negative results have been reported in double-blind placebo-controlled studies involving autologous HSC transplantation [6,7,8].

We investigated the causes of this non-response. One reason for this is that microglia accumulate because of the mixing of degenerated cells with transplanted HSCs, inhibiting re-angiogenesis [9]. In contrast, non-responders have been reported in clinical studies on the transplantation of purified HSCs [10,11]. It was considered that there were other factors in addition to the mixing of degenerated cells. Although the number of CD34 positive (CD34^+^) cells is believed to influence the efficacy of HSC therapy, there is no consensus on the optimal number of CD34^+^ cells [12,13]. Some reports have shown that the therapeutic effect on the CD34 count is dose-dependent [14,15], whereas others have shown that a low dose is effective [16] or that there is no significant difference in cell numbers [17]. The number of transplanted cells was also insufficient to account for non-responders.

In cell transplantation therapy, the “homing” ability of transplanted cells is important [18,19]. The effectiveness of the treatment is greatly influenced by whether the transplanted cells can reliably reach and act at the damaged site. Buil et al. focused on the mechanism by which leukocytes infiltrate damaged brain parenchyma during cell transplantation therapy for stroke [19]. One of the important factors in this mechanism is the adhesion factor integrin β2 (CD18). Integrins are heterodimers consisting of α- and β-chains bound in a 1:1 ratio. Among them, integrin β2 is specific to leukocytes and binds to intercellular adhesion molecule 1 (ICAM-1) on vascular endothelial cells, promoting leukocyte migration across the endothelium, including extravasation [20,21,22]. Furthermore, integrin β2 is related to the mechanism of HSC transplantation therapy for stroke that we previously revealed [23]. We demonstrated that the mechanism of HSC treatment involves the donation of metabolites as energy sources to damaged vascular endothelial cells via gap junctions, thereby rescuing damaged cells in a mouse model of stroke. It has been suggested that integrin β2 molecules work together with gap junctions [24,25,26]. Supplemental Figure S1 shows a schematic representation of the therapeutic mechanisms underlying HSC transplantation. The clinical significance of integrin β2 is well recognized. A deficiency in integrin β2 leads to leukocyte adhesion deficiency, an immunodeficiency condition characterized by refractory dermatitis and gingivitis [27]. Furthermore, it has been associated with a wide range of inflammation-related diseases, including colitis [28], COVID pneumonia [29], and dry eye disease [30]. Additionally, verification of CD18 expression in HSCs as a marker of therapeutic efficacy is of great clinical significance. To date, biomarker verification for responders to HSC therapy has mainly been conducted on the recipient side [31,32], and no studies have explored these markers in transplanted cells. Stem cell biomarkers other than human stem cells (HSCs) have been occasionally investigated [33,34]. If CD18 on HSCs is shown to be a marker that indicates the effectiveness of transplanted cells in HSC therapy, it will lead to the verification of optimal cell adjustment methods and prediction of therapeutic efficacy, leading to the widespread use of HSC therapy.

In this study, we focused on the expression of CD18 in transplanted cells and verified whether its levels affected stroke efficacy. This study aimed to develop a specific marker for selecting therapeutically effective HSCs. In the present study, umbilical cord blood (UCB) was used as a source of HSCs, and the expression of CD18 in CD34^+^ cells from each UCB sample was examined. Additionally, the relationship between the expression level of CD18 and its therapeutic effects was elucidated in a mouse model of stroke.

## 2. Materials and Methods

### 2.1. Ethical Declaration

UCB was collected from babies delivered normally at full term after obtaining informed consent from all mothers. All experiments were performed with the approval of the Ethics Committees of the Japanese Red Cross Society and Foundation for Biomedical Research and Innovation at Kobe. All animal experiments were approved by the Animal Care and Use Committee of the Foundation for Biomedical Research and Innovation in Kobe, Japan.

### 2.2. Preparation of UCB-Derived Mononuclear Cells

Whole UCB was then overlaid on Ficoll-Paque™ (Cytiva, Marlborough, MA, USA) at a volume ratio of 2:1 and centrifuged at 400× *g* for 30 min at 18–20 °C. After density gradient centrifugation, cells in the buffy coat were collected and washed twice (400× *g* for 5 min) with divalent-cation-free phosphate-buffered saline (PBS) (Thermo Fisher Scientific, Waltham, MA, USA). For cryopreservation, the precipitates were suspended in Bambanker (Nippon genetics, Tokyo, Japan) at 2 × 10^6^/mL, frozen at −80 °C, and stored in liquid nitrogen. When used, the frozen samples were rapidly thawed in a 37 °C water bath. All UCB-derived mononuclear cells (UCB-MNCs) used in this study were freeze-thawed cells.

### 2.3. Simultaneous Multiple Measurements of CD34^+^ Cells Rate and CD18 Antigens Expression in UCB-MNCs

For accurate cell identification and quantification, multiple UCB-MNC samples were examined simultaneously by flow cytometry. The cells were stained with BV421-conjugated anti-CD34 antibody (568758, BD Bioscience, Franklin Lakes, NJ, USA), FITC-conjugated anti-CD45 antibody (555482, BD Bioscience, Franklin Lakes, NJ, USA), 7AAD (559925, BD Biosciences, Franklin Lakes, NJ, USA), and R718-conjugated anti-CD18 antibody (752264, BD Biosciences, Franklin Lakes, NJ, USA), and analyzed using fluorescence-activated cell sorting (FACS) Lyric (BD Biosciences, Franklin Lakes, NJ, USA). HSCs were identified as CD34 positive, CD45 weakly positive, and 7AAD negative, whereas white blood cells (WBCs) were characterized as CD45 positive and 7AAD negative. The CD34^+^ cell rate was defined as the number of HSCs in WBCs. R718-conjugated anti-mouse IgG1 (566928, BD Biosciences, Franklin Lakes, NJ, USA) was used as an isotype control, and CD18 expression values were indicated as the ratio of the median to the isotype control (Table 1, Panel 1, Supplemental Figure S2).

### 2.4. Uptake of Vascular Endothelial Growth Factor by Human Umbilical Vein Endothelial Cells

To verify the activation of vascular endothelial cells by HSCs in vitro, we performed a vascular endothelial growth factor (VEGF) uptake experiment in a co-culture of human umbilical vein endothelial cells (HUVECs) and HSCs. HUVECs (Kurabo, Osaka, Japan) were cultured according to the manufacturer’s protocol, and passage six HUVECs were used in this study. The uptake of recombinant human VEGF (rhVEGF; R&D Systems, Minneapolis, MN, USA) was assessed using a previously described method [23,35]. In brief, biotin-conjugated rhVEGF was incubated with streptavidin-conjugated APC (Becton Biosciences, Franklin Lakes, NJ, USA) at a molecular ratio of 4:1 for 10 min at room temperature. The CD34 positivity rate in UCB-MNCs was measured in advance, and CD34^+^ cells in UCB-MNCs and the same number of HUVECs were co-cultured at 37 °C for 2 h with APC-labeled rhVEGF (at a final concentration of 10 nM). After co-culture, the cells were stained with PE-conjugated CD31 antibody (555446, BD Biosciences, Franklin Lakes, NJ, USA), FITC-conjugated anti-human CD45 antibody (555482, BD Biosciences, Franklin Lakes, NJ, USA), and 7-AAD (559925, BD Biosciences, Franklin Lakes, NJ, USA). The median APC fluorescence in CD31-positive, CD45-negative, and 7AAD-negative HUVECs was assessed using FACS, according to the same protocol [23,36].

### 2.5. Stroke Model

A stroke model with excellent reproducibility was used in 5-week-old male SCID mice (CB-17/lcr-scid/scidJcl; Oriental Yeast, Tokyo, Japan), as described previously [36,37]. Briefly, permanent focal cerebral ischemia was induced by permanent ligation and disconnection of the distal portion of the left middle cerebral artery using bipolar forceps under isoflurane inhalation anesthesia (3% for induction and 2% for maintenance). All prepared mice underwent successful surgery.

### 2.6. Cell Administration

The CD34 positivity rate and CD18 expression levels were measured in advance by flow cytometry. Different sample populations were used for in vivo immunohistochemical analysis, rotarod tests, and in vitro VEGF uptake assays because the number of cells was not sufficient to perform multiple tests. For in vivo immunohistochemical analysis, CD34^+^ cells that showed about 11 or 31 fluorescence intensity ratios of CD18 were used as low or high CD18 cells, respectively. UCB-MNCs suspended in Bambanker were injected intravenously (i.v.) 48 h after middle cerebral artery occlusion (MCAO) insult using 31G needles. The number of MNCs administered was 1 × 10^5^ for immunohistochemical analysis and 0.4 × 10^5^ for the rotarod test.

### 2.7. Immunohistochemistry

For immunohistochemical analysis, mice were anesthetized with sodium pentobarbital and perfused transcardially with saline, followed by 2% paraformaldehyde. To analyze the cortex histochemically, coronal sections (20 µM) were prepared using a vibratome (Leica, Wetzlar, Germany) by the same protocol previously [23]. The sections were immunofluorescently stained with primary antibodies against CD31 (dilution 1:50; 550274; BD Biosciences, Franklin Lakes, NJ, USA) and ICAM-1 (dilution 1:1000; 10020-1-AP Proteintech, Rosemont, IL, USA). Alexa Fluor 488- and 555-coupled antibodies (1:500; Thermo Fisher Scientific, Waltham, MA, USA) were used as secondary antibodies to visualize ICAM-1 and CD31, respectively. Nuclei were stained with 4′,6-diamino-2-phenylindole (DAPI) (dilution 1:1000; Thermo Fisher Scientific, Waltham, MA, USA). Microscopic investigations were performed using an LSM980 confocal microscope (Carl Zeiss, Oberkochen, Germany). To measure the number of blood vessels, CD31 was visualized using 3,3′-diaminobenzidine (DAB, D5637, Sigma–Aldrich, St. Louis, MO, USA) and counterstained with Mayer’s hematoxylin solution (FUJIFILM Wako Pure Chemical Corporation, Osaka, Japan). Microscopic examination was performed using a BZ-X800 microscope (KEYENCE, Osaka, Japan). Three coronal sections were selected for blood vessel counting: one at the bregma and the other two 320 µM anterior and posterior to the bregma. Parallel lines were drawn on a coronal section at positions 250, 500, 750, and 1000 µM from the top. In the peri-infarct area, the number of blood vessels with a diameter of 4 µM or more that crossed these lines and were located within 100 µM on both sides of the ischemic boundary was calculated. In the infarct core, the number of blood vessels located within 500–700 µM of the ischemic boundary was calculated (Supplemental Figure S3).

### 2.8. Rotarod Test

Sensorimotor skills were evaluated using the rotarod test. One week after cell treatment, the rotarod drum was accelerated from 4 to 40 rpm for 5 min (Muromachi Kikai Co., Ltd. Tokyo, Japan). After five weeks of cell therapy, the rotarod drum was run at a uniform speed of 30 rpm without acceleration. Each mouse was then placed on a stationary drum. The rotor was then switched on after five seconds. The time required for the mouse to fall off the rotating drum was recorded. This trial was repeated five times at 1 min intervals. The average time from five falls off the drum was used for statistical analysis.

### 2.9. Quantitative Measurements of CD18 Expression in CD34^+^ Cells by QuantiBRITE System

To quantify the CD18 expression level in CD34^+^ cells, the QuantiBRITE system (BD Biosciences, Franklin Lakes, NJ, USA) was used [38]. A phycoerythrin-labeled CD18 antibody suitable for antigen quantification with a fluorescein/protein ratio of 1:1, prepared using mouse anti-human CD18 (clone: L130), was obtained from BD Biosciences. Thawed UCB-MNCs were stained with FITC-conjugated anti-CD45 antibody (555482, BD Biosciences, Franklin Lakes, NJ, USA), 7AAD (559925, BD Biosciences, Franklin Lakes, NJ, USA), APC-conjugated anti-CD34 antibody (555824, BD Biosciences, Franklin Lakes, NJ, USA), and custom-made PE-conjugated anti-CD18 antibody (Table 1, Panel 2), and washed with PBS containing 1% fetal bovine serum. The samples were analyzed using FACSLyric. BD Quantibrite PE beads (340495, BD Biosciences, Franklin Lakes, NJ, USA) were measured using the equipment settings configured earlier, and a calibration curve was created. The CD18 expression level was defined as the number of PE molecules, that is, the number of bound antibodies, from the calibration curve. Three measurements were performed on five samples to investigate the daily variation. The coefficient of variation (CV) was calculated from the results of the three measurements.

### 2.10. Data Analysis

Statistical analyses were performed using Prism 9 software (GraphPad Software, San Diego, CA, USA). The number of blood vessels and results of the rotarod test were assessed using one-way analysis of variance with Dunn’s test. The results are expressed as mean ± standard deviation (SD). The correlation between the number of CD34^+^ cells and CD18 expression was analyzed by linear regression analysis.

## 3. Results

### 3.1. Association Between CD18 Expression in CD34^+^ Cells and VEGF Uptake Capacity of HUVECs

**Figure 1:**
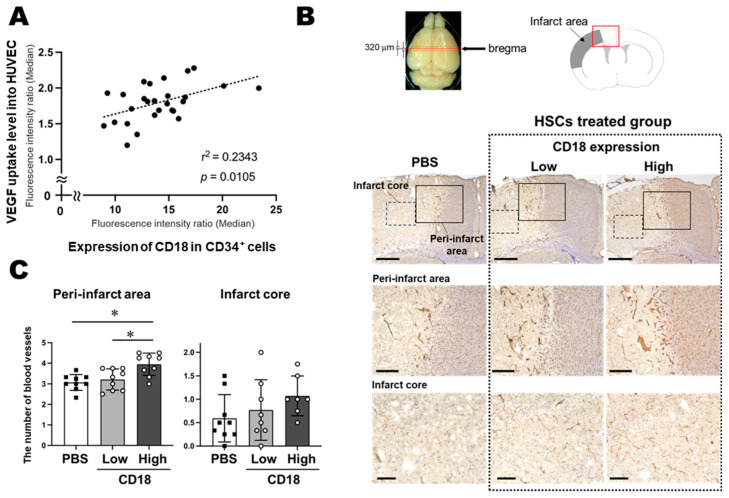
(**A**) VEGF uptake levels into HUVECs were positively correlated with CD18 expression levels in co-incubated CD34^+^ cells. (**B**) Microvasculature in the peri-infarct area and infarct core 24 h after cell transplantation. The red box in the illustration shows the immunostained area in the upper figure. (**C**) Quantitative analysis revealed that transplantation of UCB-MNCs with high CD18 expression in CD34^+^ cells reduced the degradation of the microvasculature in the peri-infarct area. * *p* < 0.05. *n* = 27 (A), *n* = 9 (**C**). Scale bars, 500 µM (upper), 200 µM (middle; peri-infarct area), and 100 µM (lower; infarct core) (**B**).

In a previous study, we observed a significant increase in VEGF uptake by HUVECs co-cultured with MNCs, including CD34^+^ cells [23]. This increase indicated the potential effect of co-cultured HSCs on the promotion of vascular endothelial proliferation. VEGF uptake into HUVECs depended on the number of CD34^+^ cells in the co-cultured MNCs; no VEGF uptake was observed in HUVECs co-cultured with CD34^+^ cell-depleted MNC [35]. In the present study, we used this assay to co-culture HUVECs with 27 individual UCB-MNC samples to examine the effects of differences in CD18 expression in CD34^+^ cells on the uptake ability of HUVECs. There was a significant positive correlation between VEGF uptake into HUVECs and CD18 expression levels in the 27 co-cultures examined (Figure 1A). This suggests that CD18 expression in CD34^+^ cells might be a key factor in promoting vascular endothelial proliferation of CD34^+^ cells.

### 3.2. Effect of CD18 Expression in Transplanted CD34^+^ Cells on Microangiogenesis in the Infarct Area

Next, the effect of CD18 expression was examined in the MCAO mouse model. UCB-MNCs containing CD34^+^ cells were i.v. injected (1 × 10^5^ cells in 100 µL PBS/mouse) into the MCAO mouse model 48 h after the insult. The number of cerebral microvessels in the infarct area (infarct core and peri-infarct area) was calculated 24 h after cell treatment. In mice transplanted with high-CD18 cells, the number of cerebral microvessels in the peri-infarct area was significantly higher than that in the PBS or low-CD18 groups. Although no significant differences were observed in the infarct core area, the high-CD18 group tended to have more microvessels than the control and low-CD18 groups (Figure 1B,C). Similar to the in vitro results shown in Figure 1A, these findings support the idea that CD18 expression in transplanted CD34^+^ cells affects the therapeutic efficacy.

### 3.3. ICAM-1 Expression in Cerebrovascular Endothelial Cells 48 h After MCAO Insult

**Figure 2:**
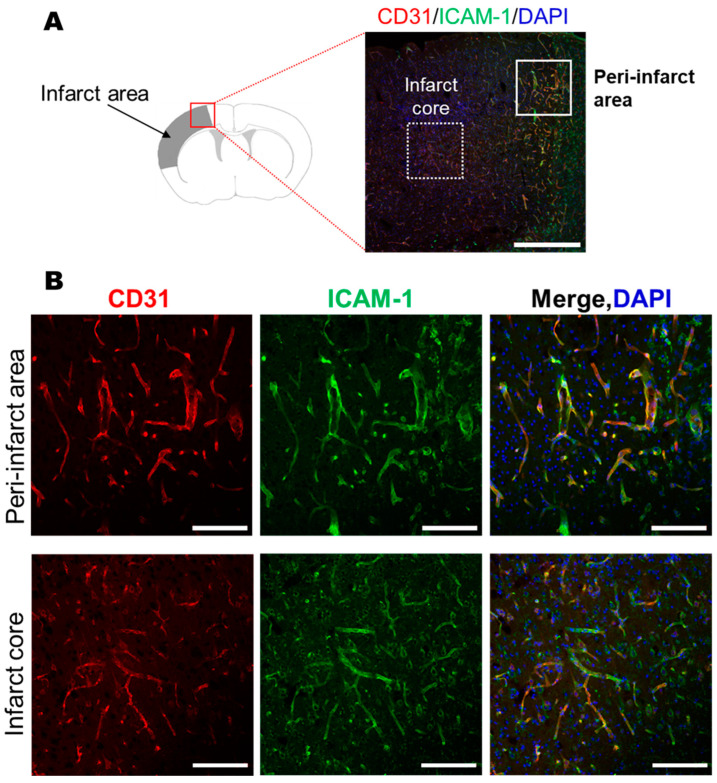
Expression of ICAM-1 was observed in the CD31-positive microvasculature in the peri-infarct area and infarct core 48 h after induction of stroke (**A**,**B**). Scale bars = 500 µM (**A**) and 100 µM (**B**). ICAM-1: intercellular adhesion molecule 1.

Using the MCAO mouse brain, immunostaining was performed 48 h after the MCAO insult, which is the optimal time for transplantation of HSCs. The expression of ICAM-1, a ligand for CD18, increased in cerebral vascular endothelial cells in the infarct area (Figure 2). This result also supports the idea that there is a high possibility that CD18 expression in CD34^+^ cells is related to the therapeutic effect of HSCs.

### 3.4. Correlation Between UCB-MNC CD34^+^ Cells Rate and CD18 Expression in CD34^+^ Cells

**Figure 3:**
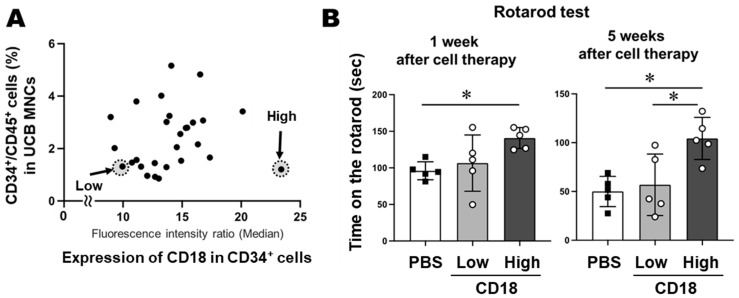
(**A**) No correlation was observed between the CD34^+^ cell rate in UCB-MNCs and CD18 expression in CD34^+^ cells. UCB-MNCs of “Low” and “High” were transplanted into mice for the rotarod test. (**B**) Mice that received “High” CD18 showed significant improvement in the rotarod test compared to the PBS control. *n* = 27 (**A**) *n* = 5 (**B**). * *p* < 0.05 (**B**).

CD18 expression in CD34^+^ cells varied significantly among UCB-MNC samples. There was no correlation between the proportion of CD34^+^ cells in UCBs and the expression level of CD18 in CD34^+^ cells in UCB-MNCs (Figure 3A). Thus, MNCs with high CD34^+^ cell content do not necessarily express high levels of CD18. This finding indicates that the number of HSCs alone may not be a reliable predictor of therapeutic effects.

### 3.5. CD18 Expression Level of HSCs and the Therapeutic Effect on MCAO MODEL Mice

CD34^+^ cells with low or high CD18 expression (Figure 3A) were administered to MCAO model mice, and the therapeutic effects of these two samples were compared. To accurately verify the effect of CD18 expression, samples with the same CD34 positivity rate but differing CD18 expression levels were selected, ensuring that the number of HSCs in the transplanted cells was approximately the same. Each sample contained 0.4 × 10^5^ MNCs in 100 µL of PBS and was i.v. injected. The low CD18 expression sample contained 419 CD34^+^ cells, and the high CD18 expression sample contained 456 CD34^+^ cells. There was a 2.9-fold difference in CD18 expression in CD34^+^ cells between the two samples (see the comparison of the median values in Supplemental Figure S4). The therapeutic effect was evaluated using the rotarod test at 1 and 5 weeks after cell treatment (Figure 3B). In the group treated with low CD18 expression, some mice showed no improvement, particularly after 5 weeks. In contrast, all mice in the group treated with high CD18 expression showed significant improvement. This result indicated that the therapeutic efficacy of CD34^+^ cells also depends in CD18 expression rather than the cell number alone.

### 3.6. Quantification of CD18 in CD34^+^ Cells

CD18 expression levels in CD34^+^ cells from five UCB-MNC samples were measured using the QuantiBRITE system. Approximately 500 CD34^+^ cells per sample were analyzed. The %CV was calculated from the three assays to verify the variability between assays. As a result, the %CV ranged from 0.38 to 3.97, with an average of 2.35, indicating that measurements were possible with little variation (Table 2).

## 4. Discussion

**Figure 4:**
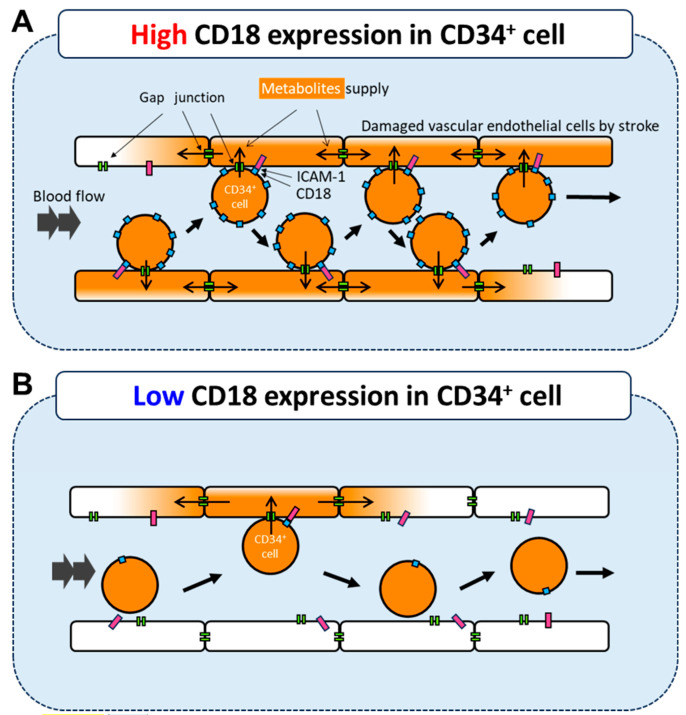
CD34^+^ cell with high CD18 expression binds to ICAM-expressing endothelial cells and provides metabolites via gap junctions (**A**). In contrast, CD34^+^ cell with low CD18 has less chance to bind to ICAM-expressing endothelial cells, resulting in less activation by transplanted cells (**B**).

In this study, we focused on an adhesion molecule expressed in CD34^+^ cells and examined whether the expression level of integrin β2 (CD18) is associated with the efficacy of cell transplantation therapy for cerebral infarction. We found that CD18 expression in CD34^+^ cells was related to its therapeutic effect both in vitro and in vivo. A schematic diagram of the mechanism is shown in Figure 4. Our results show that when performing cell therapy using HSCs, it is important to pay attention not only to the number of CD34^+^ cells but also to the expression levels of adhesion factors, especially integrin β2.

To date, no markers have been identified to evaluate the function of HSCs because the therapeutic mechanism of HSCs is unclear. Therefore, the number of CD34^+^ cells, which plays a major role after transplantation, has been the focus of HSC treatment. However, we have recently elucidated the therapeutic mechanisms of HSCs [23]. HSCs are in a glycolytically dominant metabolic state [39,40], and cell-cell interactions with vascular endothelial cells in the infarct area and metabolite donation via gap junctions play key roles in HSC treatment (Supplemental Figure S1). To clarify the treatment mechanism of HSCs, the present study focused on adhesion factors that are important for the initial contact between HSCs and vascular endothelial cells. This study demonstrated the significant role of CD18 expression in CD34^+^ cells, explaining why the number of transplanted CD34^+^ cells does not directly correlate with the strength of its effect [12,13]. These findings provide a novel marker for quantifying the potential of transplanted HSCs, which should be evaluated for significance in future clinical trials.

Interactions between integrins and connexins, which are gap junction components, have been reported in various cell types including leukocytes [24,25,26]. Integrin activation controls the expression of connexins and communication through gap junctions [24]. Since gap junctions play an important role in the treatment mechanism, the expression of integrin β2, which cooperates with gap junctions, is an important factor in treatment. Integrins are also involved in homing. Homing is induced by chemokines and involves multiple stages, including migration, rolling, and adhesion [19]. In this study, we focused on integrins that interact with gap junctions. However, it may be worthwhile to examine other factors.

At the optimal time for HSC treatment in MCAO model mice (subacute phase: 48 h after the insult), ICAM-1, a ligand for integrin β2, was highly expressed in vascular endothelial cells in the peri-infarct area (Figure 2). Therefore, the transplanted cells showed cell-cell interactions with damaged vascular endothelial cells expressing ICAM-1 during the pre-infarct period, resulting in a remarkable therapeutic effect. Previous studies have reported that ICAM-I expression in the microvasculature is increased in autopsied human brains after stroke, particularly in infarcted hemispheres [41]. These findings support the importance of CD18 expression in transplanted HSCs for clinical treatment.

Laskowiz et al. showed that allogeneic UCB-MNCs improved functional outcomes in ischemic stroke patients in (phase I trial) [8]. However, they did not show any benefits in a phase II trial [42]. The use of allogeneic cells makes it possible to select cells for transplantation in advance. If the transplanted cells had been selected based on their involvement in the therapeutic mechanism, outcome improvement might have been achieved in a phase II trial. This lack of response may be attributable to the low expression of CD18 in the transplanted HSCs. HSCs are remarkably effective in the treatment of various ischemic diseases, and numerous clinical trials on HSCs are ongoing worldwide. Cell selection based on therapeutic mechanisms may increase the reliability of the therapeutic effects of HSCs.

This study demonstrated the importance of CD18 in the treatment of cerebral infarction. The fundamental therapeutic mechanism begins with the adhesion of HSCs to damaged blood vessels expressing ICAM-1. It has been suggested that a similar mechanism occurs in the treatment of various diseases in which blood vessels express ICAM-1. It has been reported that ICAM-1 is expressed in the blood vessels of mouse ischemic limbs [43] and in the postmortem hearts of patients with myocardial infarction [44]. Furthermore, it has been reported that in the treatment of myocardial infarction, the therapeutic effect is improved when CD18 is overexpressed in transplanted adipose-derived stem cells [45], indicating that CD18 on transplanted cells is also important in the treatment of myocardial infarction. From the above, it is strongly suggested that the findings of this study can be applied to cell therapy for various diseases.

The expression level of CD18 in CD34^+^ cells was considered a specification marker for the transplanted cells. Flow cytometry is one of the simplest and most useful methods for cell evaluation. However, a major drawback of this method is that it can only be evaluated as a relative value in each experiment. Therefore, it cannot be determined using the expression value alone. The QuantiBRITE system can quantitatively measure the amount of antigen as an absolute value with high reproducibility. It has already been used in clinical settings [46,47] and was used in this study as well. However, although the QuantiBRITE system is a highly accurate measurement method with little variability in measurements, there are differences in variability depending on the marker type [38]. In this study, we quantified a small number of frozen cells, and the average %CV value was found to be approximately 2.8%. In the future, to use it as a specification marker in clinical settings, it will be necessary to increase the number of samples and verify the variation between assays, measurers, and facilities. In addition, the differences between the instruments can be eliminated using controls; therefore, this should be used as a reference [48]. This study suggests that the expression of CD18 in CD34^+^ cells can be quantified and adequately measured in clinical settings. Furthermore, by accumulating measurement data from actual transplanted CD34^+^ cells, we aimed to verify the relationship between treatment effects and measurements to establish a specification marker.

This study has the following limitations: (1) The reason why the levels of CD18 in CD34^+^ cells are significantly varied between samples remains unsolved. While standardized procedures were used for cord blood collection, elusive differences in cord blood—such as subclinical infection during pregnancy, irritation of the coagulation cascade, or genetic polymorphisms—might affect the level of CD18 expression in CD34^+^ cells. Notably, anticoagulant factor levels have been reported to be associated with CD18 levels [49]. (2) Further investigation is needed to identify new markers other than CD18, such as adhesion factors and chemokines. (3) The importance of CD18 expression in CD34^+^ cells should be evaluated in other animal models and confirmed in clinical trials to broaden its applicability.

## 5. Conclusions

The present study demonstrates the importance of integrin β2 in HSC efficacy in an MCAO mouse model. This suggests that CD18 expression in HSCs is an important factor in the therapeutic efficacy of HSC transplantation therapy and can be used to distinguish non-responders. Furthermore, this finding might be applicable not only to cerebral infarction but also to various other ischemic diseases.

## 6. Patents

Yuko Ogawa, Kazuta Yasui, Akihiro Fuchizaki, Mitsunobu Tanaka, Takafumi Kimura, and Akihiko Taguchi have applied for a patent about highly expressed CD18 in CD34-positive cells for cell-based therapy (PCT/JP2023/007766).

## Figures and Tables

**Table 1:** Antibodies used for fluorescence-activated cell sorting (FACS) analysis.

Panel	Antibody	Fluorochrome	Manufacturer	Catalog No.
1	CD34	BV-421	BD Biosciences	568758
	CD45	FITC	BD Biosciences	555482
	7-AAD	-	BD Biosciences	559925
	CD18	R-718	BD Biosciences	752264
	Mouse-IgG1	R-718	BD Biosciences	566928
2	CD34	APC	BD Biosciences	555824
	CD45	FITC	BD Biosciences	555482
	7-AAD	-	BD Biosciences	559925
	CD18	PE	BD Biosciences	Custom made

**Table 2:** %CV of CD18 expression levels.

Sample No.	1	2	3	4	5
%CV	3.92	0.38	3.97	2.72	0.76

## Data Availability

The original contributions of this study are included in the article and supplementary materials. Further inquiries can be directed to the corresponding authors.

## Supplementary Materials

The following supporting information can be downloaded from https://www.mdpi.com/article/10.3390/life15020195/s1, Figure S1: The therapeutic mechanism of HSC transplantation; Figure S2: Flow cytometry analysis of UCB-MNCs; Figure S3: Defining boundaries for counting blood vessels; Figure S4: Flow cytometry analysis of low or high CD18 expression samples.
