# Supplementary material for: Integrin β2 Plays a Significant Role in Therapeutic Angiogenesis Through Hematopoietic Stem Cell Transplantation
Source: Life (Basel). 2025 Jan 28;15(2):195. doi: 10.3390/life15020195 (PMC11856074; doi:10.3390/life15020195)
Supplement: Supplementary file 1 [file life-15-00195-s001.zip › life-3366726-supplementary.pdf]

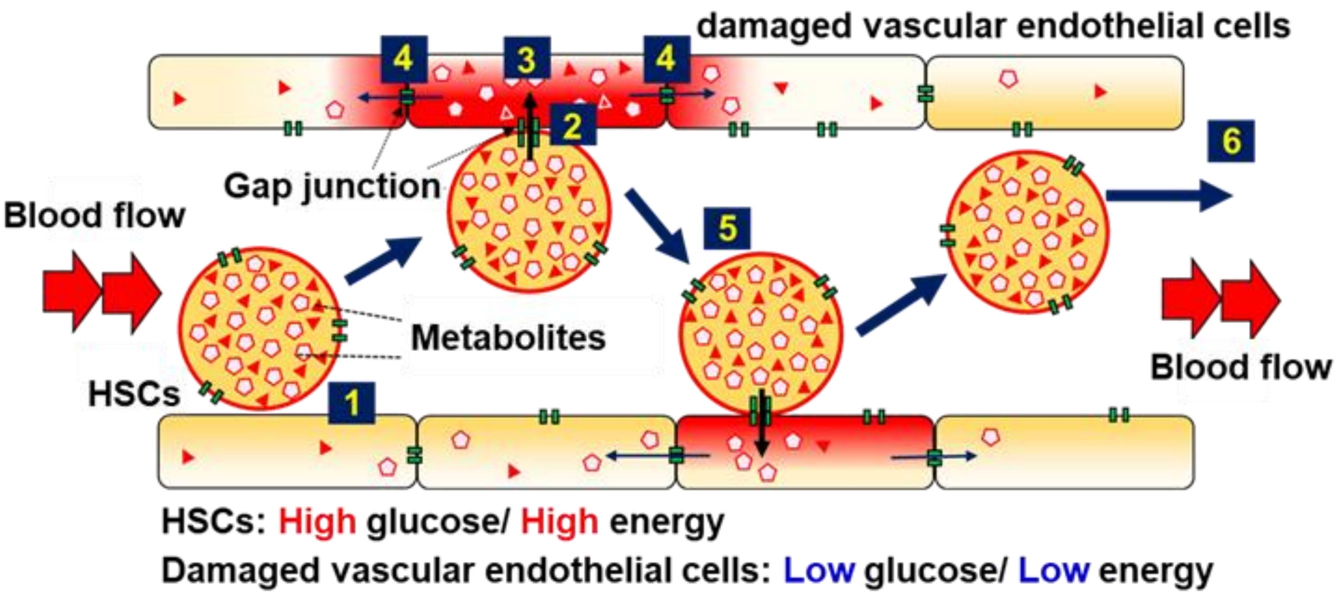

Supplemental Figure S1. The therapeutic mechanism of HSC transplantation

(1) Hematopoietic stem cells (HSCs) home to the infarct area and undergo short-term adhesion to damaged vascular endothelial cells. (2) HSCs and damaged vascular endothelial cells are connected via gap junctions. (3) Low-molecular-weight metabolites are supplied as energy sources from HSCs to damaged vascular endothelial cells. (4) Energy metabolism in the adjacent vascular endothelial cells is activated via gap junctions. (5) When the short-term adhesion ends, the HSCs adhere to another endothelial cell.

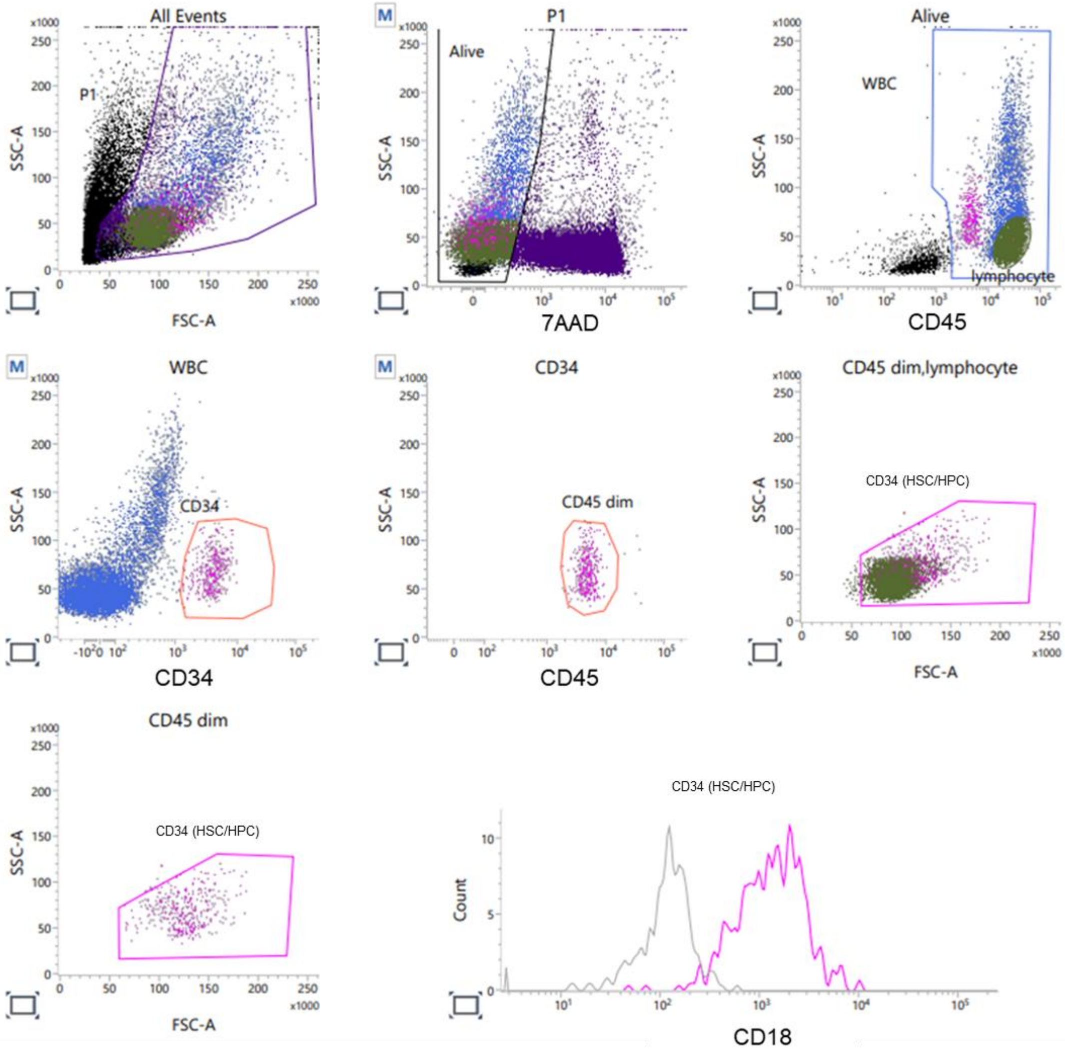

Supplemental Figure S2. Flowcytometry analysis of UCB-MNCs

In the histogram of $CD34^+$ HPCs, the pink line indicates the expression of CD18 on $CD34^+$ cells, and the gray line indicates the isotype control. HSC: Hematopoietic Stem Cell, HPC: Hematopoietic Progenitor Cell

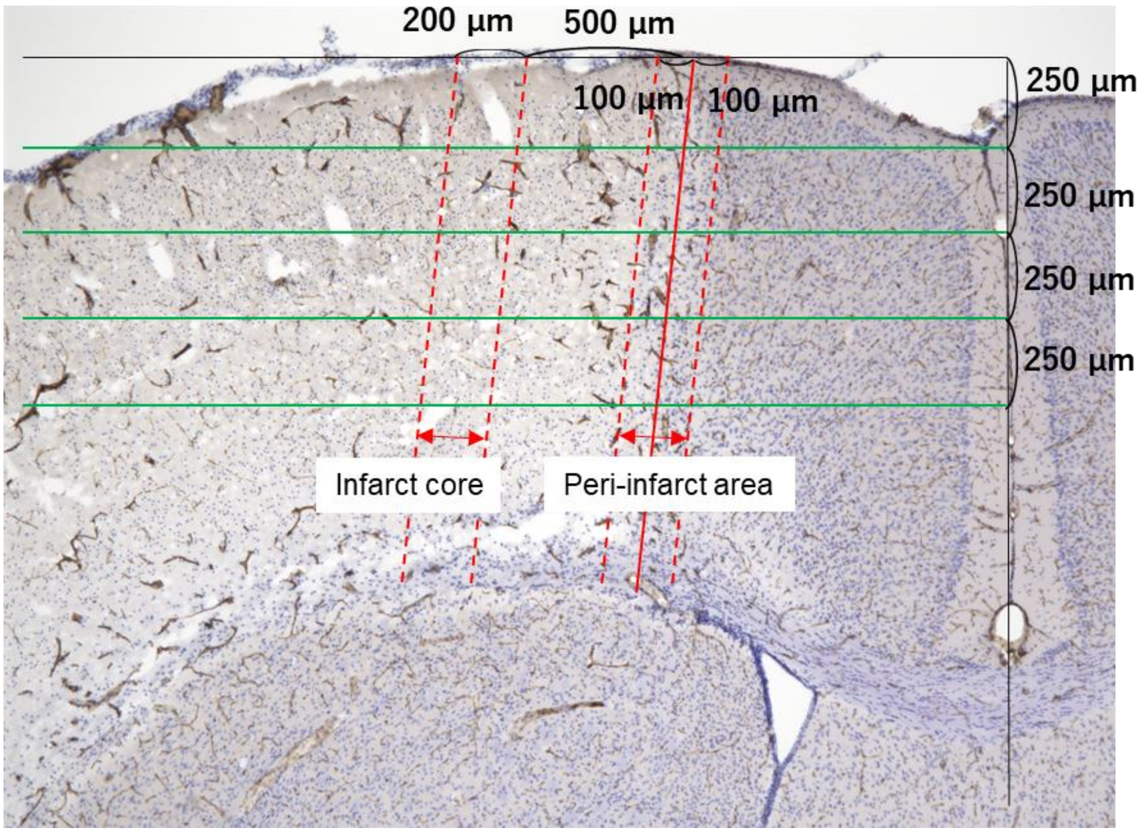

Supplemental Figure S3. Defining boundaries for counting blood vessels

Four parallel lines (green) were drawn on the coronal section at positions 250, 500, 750, and 1000 µm from the top. The ischemic boundary (red straight line) is calculated based on the shape of the nucleus. In peri-infarct area, the number of blood vessels that crossed four parallel lines and were located within 100 µm on both sides from the ischemic boundary was calculated. In infarct core, the number of blood vessels located in 500–700 µm from the ischemic boundary was calculated.

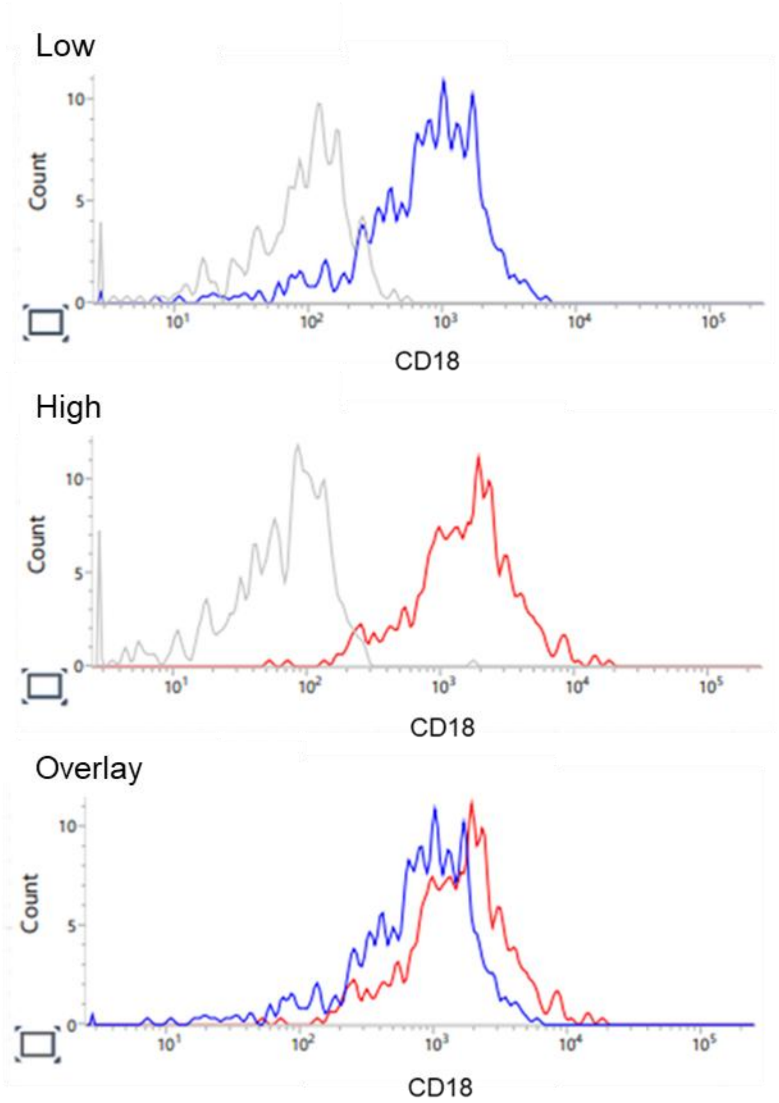

Name	Events	CD18 Median
Low: CD34 (HSC/HPC)	417	858
Low isotype: CD34 (HSC/HPC)	335	95
High: CD34 (HSC/HPC)	377	1,661
High isotype: CD34 (HSC/HPC)	453	64

Supplemental Figure S4. Flowcytometry analysis of low and high CD18 expression samples

In the histogram of CD34 (HSCs/HPCs), the colored line (blue: low CD18 expression sample, red: high CD18 expression sample) indicates the expression of CD18 on $CD34^+$ cells, and the gray line indicates the isotype control. HSC: Hematopoietic Stem Cell, HPC: Hematopoietic Progenitor Cell
